# Supplementary material for: Click chemistry-based quantification of extracellular matrix turnover for drug screening and regenerative medicine
Source: bioRxiv. 2025 May 14:2025.05.08.652928. Preprint. [Version 1] doi: 10.1101/2025.05.08.652928 (PMC12132353; doi:10.1101/2025.05.08.652928)
Supplement: Supplement 1 [file media-1.pdf]

Supplementary Material

Click chemistry-based quantification of extracellular matrix turnover for drug screening and regenerative medicine

*Annie Porter,[1] Songshan Fan,[1] Ying Peng,[1] Mengxi Lv,[1] Yilu Zhou,[1] Abdulaziz Alanazi,[2] Lin Han,[2] Liyun Wang,[1] X. Lucas Lu[1]**

[1]Department of Mechanical Engineering, University of Delaware, Newark, Delaware, 19716, USA

[2]School of Biomedical Engineering, Science and Health Systems, Drexel University, Philadelphia, PA 19104, USA

[*]*Corresponding Author, E-mail address:* xlu@udel.edu

This supporting information contains:

Table S1. Methionine Content of ECM Proteins S2
Figure S1. Illustration of ECM loss profiles for individual cartilage samples tracked by click chemistry and traditional chemical assays. S3

Table S1: Methionine content of select common ECM proteins in bovine articular cartilage. Retrieved from UniProtKB/Swiss-Prot.[1]

UniProtID	Protein Name	Gene	# of Amino Acids	# of Methionines	**Methionine Content (%)**
P02459	Collagen alpha-1(II) chain	COL2A1	1,487	20	**1.34**
C0HLN2	Collagen alpha-2(IX) chain	COL9A2	688	10	**1.45**
Q28083	Collagen alpha-1(XI) chain	COL11A1	911	10	**1.10**
Q32S24	Collagen alpha-2(XI) chain	COL11A2	1,736	19	**1.09**
P13608	Aggrecan core protein	ACAN	2,364	10	**0.42**
P35445	Cartilage oligomeric matrix protein	COMP	756	11	**1.45**

[1] The UniProt Consortium. UniProt: The Universal Protein Knowledgebase in 2023. *Nucleic Acids Res.* **2023**, *51* (D1), D523–D531. https://doi.org/10.1093/nar/gkac1052.

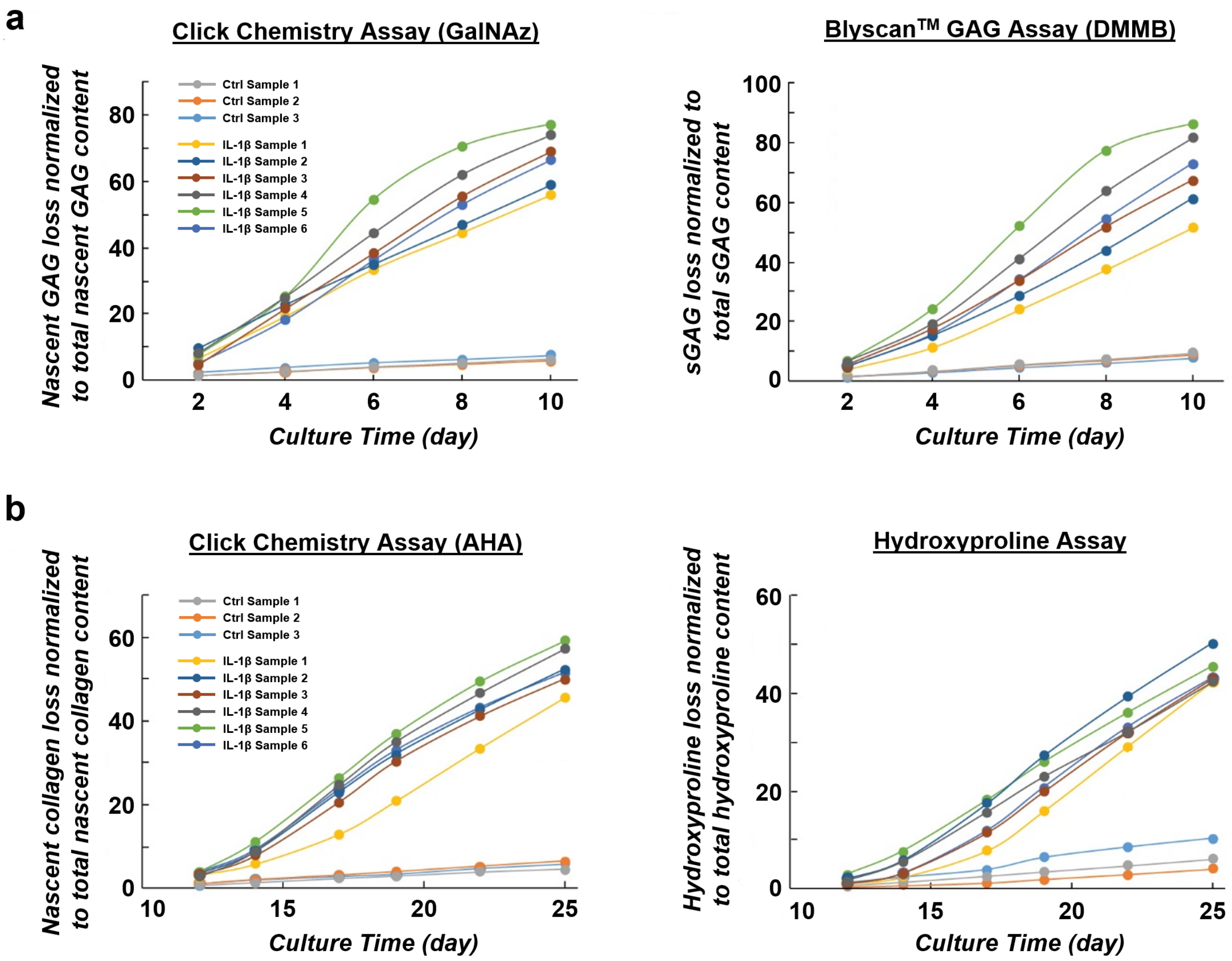

Figure S1. Illustration of ECM loss profiles for individual cartilage samples tracked by click chemistry and traditional chemical assays. Illustrated here are the longitudinal ECM loss profiles for individual cartilage samples (n=3 ctrl, n=6 IL-1β). (a) The loss of glycosaminoglycans (GAG) tracked by click chemistry using a GalNAz tag and the traditional dimethylmethylene blue (DMMB) assay for sulfated GAGs. The loss of GAG into the culture media from cartilage samples exposed to the pro-inflammatory cytokine interleukin 1-beta (IL-1β; 1ng/ml) was tracked for 10 days. (b) The loss of collagen tracked by click chemistry using an AHA tag and the traditional hydroxyproline assay. The loss of collagen into the culture media from cartilage samples exposed to the pro-inflammatory cytokine IL-1β (1 ng/ml) was tracked for 25 days.
